# Preclinical Development of Mahanine-Enriched Fraction from Indian Spice *Murraya koenigii* for the Management of Cancer: Efficacy, Temperature/pH stability, Pharmacokinetics, Acute and Chronic Toxicity (14-180 Days) Studies

**DOI:** 10.1155/2020/4638132

**Published:** 2020-08-10

**Authors:** Eswara Murali Satyavarapu, Prasun Kumar Sinha, Chitra Mandal

**Affiliations:** Cancer Biology and Inflammatory Disorder Division, Council of Scientific and Industrial Research-Indian Institute of Chemical Biology, 4, Raja S.C. Mallick Road, Kolkata 700032, India

## Abstract

*Murraya koenigii* is well documented in the Indian ancient medical text “Charaka Samhita.” The carbazole alkaloid “mahanine” from this plant exhibited anticancer activity against several cancers. Here, we have taken a comprehensive study to standardize the method for the preparation of a mahanine-enriched fraction (MEF) with the highest yield and defined markers. Our optimized method produced MEF having the highest amount of mahanine, a major marker, with excellent *in vitro* antiproliferative activity against ovarian and breast cancer cells as evidenced by decreased cell viability by MTT assay. Moreover, it exhibited condensed and fragmented nuclei by DAPI staining and increased annexin V-/PI-stained cells after MEF treatment, indicating apoptosis. It also exhibited good efficacy in ovarian and breast cancer syngeneic mice models, with an ED50 of 300 mg/kg body weight (BW). MEF is stable up to 40°C for ≥3 months. Its biological activity remains unchanged at a wide range of pH (1-10) for up to ~3 hours, indicating a safe oral route of administration. Additionally, the comparative pharmacokinetics of MEF and mahanine in rats showed a 31% higher bioavailability of mahanine in MEF-fed rats compared to rats fed with mahanine alone. Furthermore, mice fed with MEF at 5000 mg/kg BW single dose, 300-1500 mg/kg BW/day for 14 days, and 300 mg/kg BW/day for 28, 90, and 180 days for subacute, subchronic, chronic studies, respectively, did not show any significant clinical signs of toxicity, behavioral changes, mortality, organ weights, serum biochemistry, and hematological parameters indicating no/minimum toxicity for up to 180 days. To the best of our knowledge, this is the first report showing the pH/temperature stability and chronic toxicity studies of MEF along with *in vivo* efficacy against breast cancer. Taken together, our study will enhance the commercial value of this highly potential medicinal plant and will be helpful as a reference material for its clinical development.

## 1. Introduction

Phytopharmaceutical or plant-derived natural products are vital medicinal sources globally and are considered an important alternative to modern allopathic medicine for many diseases because of their health benefits and lower cost [1]. However, the safety of herbal medicines remains a major concern because of the lack of quality control using specific well-characterized chemical markers and long-term toxicity evaluation [2]. Therefore, different countries are focusing on promoting the safety, efficacy, and quality of herbal drugs. The World Health Organization (WHO) introduced the Traditional Medicine (TM) Strategy 2014–2023. India has also introduced Appendix I-B to Schedule Y for the regulation of phytopharmaceuticals [3]. Additionally, the biological activity of plant extracts is mainly due to secondary metabolites like alkaloids, flavonoids, and terpenoids. The plant produces secondary metabolites mainly for defense against microbes and environmental stress. Consequently, their concentration is highly dependent on environmental factors like photoperiod, light intensity/quality, temperature, soil, and water [4]. Therefore, different techniques like extraction with varying compositions of solvents or by fractionation using two immiscible solvents or both will be very helpful for the preparation of better phytopharmaceuticals with an enhanced concentration of biologically active molecules.

*Murraya koenigii* (L.) Spreng. leaves, commonly called curry leaves or Kari patta, are used as a spice in the day-to-day routine of Indian people, especially in the southern part of India, and is therefore expected to be nontoxic. This Indian medicinal plant belongs to the family *Rutaceae.* It has been well documented in *Charaka Samhita*, an encyclopedia of Ayurveda. It is reported to have many therapeutic uses for different diseases [5]. However, as any effective drug may produce adverse drug reactions and herbal medicines are of no exception, the long-term toxicity (90 and 180 days) of this plant extract needs to be investigated.

This plant is a rich source of carbazole alkaloids; in the last ten years, we have isolated and extensively characterized a biologically active carbazole alkaloid (mahanine) from the leaflets of this plant which showed both *in vitro* and *in vivo* antiproliferative activity against many different cancer cell lines and animal models which include leukemia, pancreatic cancers, and glioblastoma, with various mutations [6]. It is a prooxidant molecule [7, 8] that inhibits the mammalian target of rapamycin (mTOR)C1/2 in glioblastoma multiforme [9], induces microtubule-associated protein 1A/1B light chain 3B- (LC3-) mediated anoikis, and inhibits autophagy in ovarian cancer [10]. Additionally, it showed a good synergistic effect with clinically approved drugs both in colon and cervical cancers [11, 12]. Moreover, it exhibited antileishmanial activity through immunomodulation [13].

In our previous study, we have systematically optimized the geographical location and season for the collection of this valuable plant with the highest mahanine content and prepared a fraction rich in mahanine with two more defined markers like mahanimbine and koenimbine [14]. Additionally, this fraction exhibited antiproliferative activity in both *in vitro* in sixteen different cancer cell lines of nine types of cancer and *in vivo* in ovarian and lung cancer xenograft mice models [14]. However, the efficacy of the fraction rich in mahanine was not tested in syngeneic ovarian and breast cancer models.

Therefore, the purpose of the study is to develop a mahanine-enriched fraction (MEF) as a novel phytopharmaceutical for the management of cancer by generating all necessary preclinical data which includes anticancer efficacy, stability, acute and chronic toxicities, and pharmacokinetics for getting approval from the regulatory bodies for its clinical trials.

Encouraged by these observations, to further improve the MEF preparation method, here, we compared five different methods to optimize the yield of mahanine content. The selected MEF is stable even at 40°C under darkness/light in glass/polypropylene containers for ≥3 months and with the potential of hydrogen (pH: 1-10) up to ~3 hours (h) without losing its biological activity. MEF is nontoxic at a repeated oral dose (1000 mg/kg body weight (BW)/day) for 14 days in mice. It induced apoptosis in cancer cells as evidenced by condensed and fragmented nuclei by DAPI staining and increased annexin V-/PI-stained cells after MEF_M2_ treatment. Additionally, it reduced tumor mass in both ovarian and breast cancer syngeneic mice models. Orally fed mice for 14-180 days at an ED50 (effective dose) dose did not show any clinical signs of toxicity (blood/organs), behavioral changes, or mortality. Taken together, this optimized mahanine-enriched fraction with a high-pressure liquid chromatography (HPLC) fingerprint for quality assessment, stability, *in vitro* and *in vivo* efficacy, and long-term toxicity studies will be helpful for the preparation of the best phytopharmaceutical for the management of cancer.

## 2. Methods

### 2.1. Materials

Cell culture medium, Roswell Park Memorial Institute- (RPMI-) 1640, minimal essential medium Eagle's (MEM), fetal bovine serum (FBS), antibiotic-antimycotic (containing penicillin, streptomycin, and Gibco Amphotericin B), and trypsin-ethylene diamine tetraacetic acid (EDTA) were from Invitrogen. 3-(4,5-Dimethylthiazol-2-yl)-2, 5-diphenyl tetrazolium bromide (MTT), dimethyl sulphoxide (DMSO), and matrigel were from Sigma-Aldrich, USA. Ethanol (Spectrosol E, HPLC grade ≥ 99%; CAS# 64-17-5) was purchased from Spectrochem Pvt. Ltd., India. Ethyl acetate (HPLC grade ≥ 99%) was purchased from Merck, India.

### 2.2. Cell Cultures

Human ovarian (PA1 and OVCAR3) and breast (MCF7) cancer cell lines were purchased from the NCCS Cell Repository, Pune. The mouse ovarian cancer (ID8) cell line was a kind gift from Dr. Sib Sankar Roy, CSIR-Indian Institute of Chemical Biology (IICB), Kolkata, and the mouse breast cancer cell line 4T1 was a gift from Dr. Dipyaman Ganguly, CSIR-IICB, Kolkata. All cells were grown in the respective medium as mentioned in Table 1, supplemented with 10% FBS, glutamine (2.2 g/L), and 1% antibiotic-antimycotic (complete medium) at 37°C with 5% carbon dioxide.

### 2.3. Collection and Drying of Plant Material

We have earlier optimized a place and season for the collection of leaflets from the *Murraya Koenigii* plant having the highest amount of mahanine [14]. Accordingly, we procured the leaflets from Tamil Nadu in India (13.0675°N, 80.1951°E) between September and December. The voucher specimen was identified by Dr. Debabrata Maity, Department of Botany, Kolkata (20033, CUH). These fresh leaflets (500 grams) were washed and dried at 30-35°C until the leaflets become brittle (~100 grams). They were crushed into small pieces and stored in airtight containers at 4°C for further use.

### 2.4. Preparation of the Mahanine-Enriched Fraction

#### 2.4.1. Method 1

Dry leaflets (50 grams) were infused with hot distilled water (1000 mL, 50°C) for four hours with occasional stirring. The water portion was filtered and discarded as mahanine is insoluble in water. The remaining residue was dried and macerated with ethanol (~1000 mL) at ~30°C for 72 hours. This ethanolic extract was fractioned with ethyl acetate (100 mL) and water (50 mL) in a 2 : 1 ratio, and the organic layer was dried, weighed, and named as mahanine-enriched fraction MEF_M1_. Similar method was previously reported by our group, where water infusion of leaflets followed by ethanolic extraction was only done without a fractionation step [14].

#### 2.4.2. Method 2

Similarly, leaflets (50 grams) were infused with hot distilled water and the remaining residue was dried and macerated with 1000 mL ethanol : water (95 : 05) at ~30°C for 72 h. The dried extract was weighed and fractionated with ethyl acetate and water. The ethyl acetate fraction was collected, dried, and weighed, and then it was designated as MEF_M2_.

#### 2.4.3. Method 3

Leaflets (50 grams) were directly macerated with ethanol : water (95 : 05). This extract was fractioned with ethyl acetate and water and processed similarly, then it was named MEF_M3_.

#### 2.4.4. Method 4

Leaflets (50 grams) were similarly macerated with ethanol : water (90 : 10). The ethanolic extract was dried and weighed. This extract was similarly fractioned with ethyl acetate and water. This fraction was named MEF_M4_.

#### 2.4.5. Method 5

Leaflets (50 grams) were macerated with ethanol : water (80 : 20). This extract was fractioned similarly and named MEF_M5_.

All the final products were completely dried using a Lyophilizer (Martin Christ Alpha 1-2 LDplus freeze dryer, with a temperature of -55°C and with a high vacuum, ice condenser performance of 2 kg/24 hrs) for 6 hours/day for 3 days to remove any traces of solvents and were stored at -20°C for further analysis.

### 2.5. HPLC Analysis

All extracts from Methods 1 to 5 were dissolved in the respective maceration solvents, and the final products (MEF_M1-5_) were dissolved in ethanol to make 10 mg/mL stocks. A substock (500 *μ*g/mL) was prepared with methanol : water (80 : 20) and filtered. A sample (50 *μ*L) was injected into an HPLC (Waters 2487 Dual *λ* Absorbance UV Detector; 254 nm) and Reverse-Phase C18 column (5 *μ*m, 250 × 4.6 mm; Waters, USA) and run in an isocratic solvent system (methanol : water (80 : 20)) with a 1.0 mL/minute flow rate. Mahanine percentage was calculated by the external calibration method using EMPOWER 2 software and compared [15].

### 2.6. MTT Assay

MEF_M2_ was dissolved in ethanol (10 mg/mL), and the substock was prepared with complete medium. The antiproliferative activity was checked against PA1, OVCAR3, ID8, MCF7, and 4T1 cell lines. Cell viability was determined by MTT assay. Cells (4 × 10^3^, 250 *μ*L/well) were separately treated with different concentrations of MEF_M2_ (0–40 *μ*g/mL) in triplicate in a 96-well plate and incubated for 48 h at 37°C. The medium was discarded, and MTT (100 *μ*g/well in IMDM) reagent was added and incubated for 3 h at 37°C. Formazan crystals formed by the live cells were dissolved in DMSO. The intensity of the color was quantified at 550 nm in a 96-well plate reader,and the concentration of MEF_M2_ that induced 50% cell death (half maximal inhibitory concentration; IC50) was calculated.MEF_M2_ aliquots were kept at different temperatures, pH, and light conditions in different containers were also checked similarly for their biological activity in PA1 cells for comparison.

### 2.7. Nuclear Staining with DAPI

PA1 (5 × 10^5^ cells/well) was treated with MEF_M2_ and fixed with 4% paraformaldehyde (PFA). Cells were washed, stained with 4,6-diamidine-2-phenylindolehydrochloride (DAPI, 1 mg/mL) in methanol for 1 min at 25°C, washed thrice with PBS, and visualized using a Leica DM 6000B microscope. Others cells were also similarly processed.

### 2.8. Annexin V/PI Positivity

PA1 cells (5 × 10^5^/well) were incubated with different concentrations of MEF_M2_ for 24 h at 37°C. These cells were trypsinized, centrifuged, washed, and suspended in annexin V binding buffer and then incubated for 45 min in the dark at 25°C. Annexin V-FITC/PI (1 *μ*g/mL) was added and kept for 20 min in the dark at 4°C [10]. These stained cells were analyzed by FACS with CellQuest Pro software. OVCAR3 cells were also treated with MEF_M2_ and processed similarly.

### 2.9. Temperature Stability of MEF_M2_

An equal amount (1.0 mg/100 *μ*L) of MEF_M2_ was distributed into five glass vials and two polypropylene centrifuge tubes and kept at different temperatures, without or with light exposure for 90 days. All these samples were analyzed for their antiproliferative property against PA1 by MTT assay and compared with freshly prepared MEF_M2_.

### 2.10. pH Stability of MEF_M2_

The stability of MEF_M2_ at a wide range of pH (1-10) was analyzed. Hydrochloric acid (HCl; 0.1 N) solution, sodium bicarbonate buffer + HCl, phosphate-buffered saline (PBS), and sodium bicarbonate buffer with a pH of 1.0, 5.0, 7.2, and 10.0 were used, respectively [16]. An equal amount of MEF_M2_ was taken in these buffers separately and incubated at 37°C for 2 or 3 hours on a shaker. pH was neutralized at ~7.0 and extracted with an equal volume of ethyl acetate. They were dried and processed for HPLC analysis.

### 2.11. Grouping of Animals

Male and female Balb/c mice (6-8 weeks old) and female Sprague-Dawley rats (8-10 weeks) were obtained from the animal house facility of CSIR-IICB. All animals were kept in 12 h light/dark cycles with standard diet and water. Animals were acclimated for 7 days. Four mice/cage and two rats/cage were kept in individually ventilated cages.

#### 2.11.1. Method of Animal Sacrifice

All animals were sacrificed by euthanasia with diethyl ether anesthesia (2% ether on a cotton pad in a conical tube for ~5 minutes) followed by cervical dislocation.

#### 2.11.2. Ethics Statement

This study has been approved by the institutional animal ethics committee (CSIR-IICB-AEC) on animal experimentation (Ref No. IICB/AEC/Meeting/2016/October; dated: 31.10.2016).

### 2.12. *In Vivo* Syngeneic Mice Models

MEF_M2_ (1.0 gram) was dissolved in ethanol (6.0 mL), diluted with sterile double-distilled water (34 mL) to make a uniform suspension, and used for oral feeding. 15% of the ethanol in water was fed to untreated control mice.

Female Balb/c mice (*n* = 10/group) were injected subcutaneously with ID8 and 4T1 cancer cells (5 × 10^6^) in 100 *μ*L matrigel : RPMI-1640 (1 : 1) separately to generate ovarian and breast cancer models, respectively. Tumors (~100 mm^3^) were generated within 5-7 days. Animals were fed orally with MEF_M2_ at a dose of 300 mg/kg BW/day for 14 days in the ovarian cancer (OC) model and 20 days in the breast cancer model. Additionally, breast cancer mice were fed orally with MEF_M2_ at doses of 600 and 900 mg/kg BW/day separately to check for any possible loss in body weight. In a positive control group, paclitaxel (10 mg/kg BW, intravenous administration) was used in representative breast cancer mice. The tumor size and body weight were monitored.

### 2.13. Comparative Pharmacokinetic Studies between MEF_M2_ and Its Main Biologically Active Carbazole Alkaloid, Mahanine

Adult rats were fasted overnight and divided into two groups (*n* = 3). One group of rats was fed orally with MEF_M2_ 300 mg/kg BW/day (per os (P.O.)). Similarly, the second group of animals were fed with mahanine (P.O.) equivalent to 300 mg/kg BW MEF_M2_. Blood was collected from all these animals through the retroorbital plexus at 0 h (predose), 0.75 h, 2.5 h, 4 h, 6 h, and 8 h. 50 *μ*L of plasma at each time point was mixed with 1 mL of ethyl acetate. The organic portion was dried and resuspended in running solvent (methanol : water, 80 : 20) and analyzed by HPLC as mentioned above. The amount of mahanine peak was calculated, and the time vs. concentration curve was plotted [17].

### 2.14. Toxicity Studies in Mice

Acute, subacute, subchronic, and chronic toxicity studies were performed according to the guidelines of the Organization for Economic Cooperation and Development (OECD) 420, 407, 408, and 452, respectively. Female mice were used for acute and subacute (28 days) toxicity studies, and both female and male mice were used for subchronic (90 days) and chronic (180 days) toxicity studies.

Initially, a single dose of 2000 mg/kg BW/day and 5000 mg/kg BW/day of MEF_M2_ were administered separately in smaller fractions over a period not exceeding 24 hours by oral gavage to each animal to determine the maximum tolerated dose (MTD). Next, repeated-dose MTD mice were fed with MEF_M2_ (300, 500, 750, 1000, and 1500 mg/kg BW/day) for 14 days in an individual group of mice (*n* = 5). The mice fed with 15% ethanol in water served as vehicle control. All these mice were observed individually for signs of behavioral changes and activity for 3 h post dosing and at least once daily for 14 days. At the end of the study, serum biochemistry and histopathology were performed.

Additionally, female mice (*n* = 12) were randomly divided into two groups for a subacute (28-day) oral toxicity study. Furthermore, female (*n* = 20) and male (*n* = 20) mice were used for a subchronic (90-day) toxicity study. Moreover, for chronic (180-day) toxicity studies, female (*n* = 20) and male (*n* = 20) mice were similarly divided into two groups. Vehicle or extract (300 mg/kg BW) was orally administered daily to control and treated groups, respectively, for 28, 90, and 180 days. All these animals were observed at least twice daily for morbidity and mortality. The body weights of the animals were measured at regular intervals throughout the feeding period. Mice were sacrificed at the end of each experiment.

Blood (~0.5 mL) was collected through the retroorbital plexus. The serum was stored at -80°C until analysis. Blood urea nitrogen (BUN), urea, creatinine, gamma-glutamyl transferase (GGT), bilirubine, total cholesterol, serum glutamic oxaloacetic transaminase (SGOT), and total protein, albumin (ALB), globulin (GLOB), alanine aminotransferase (ALT), aspartate transaminase (AST), alkaline phosphatase (ALP) triglycerides, and lactate dehydrogenase (LDH) were evaluated in an automated biochemical analyzer.

Additionally, blood (~0.75 mL) was also collected into tripotassium ethylenediaminetetraacetic acid (K3 EDTA) in 2.0 mL tubes for the determination of hematological parameters. Erythrocytes, leukocytes, neutrophils, eosinophils, lymphocytes, monocytes, haemoglobin, platelets, packed cell volume, and erythrocyte sedimentation rate were analyzed. Organs (heart, liver, kidney, lungs, and spleen) were collected from acute, sub-chronic and chronic study mice, weighed, and fixed in a 10% buffered formalin solution. These fixed organs were processed for paraffin embedding, sliced, and Haematoxylin and Eosin (HE) stained, and then histomorphological observation was performed under the microscope.

### 2.15. General Behavior and Mortality

All animals were carefully examined for abnormal behavior and appearance during the acclimatization period. All mice were observed at least once a day for mortality or morbidity; changes in skin, fur, eyes, and mucous membranes; respiratory autonomic effects (e.g., salivation); central nervous system effects (tremors and convulsions); changes in the level of locomotor activity and posture; and reactivity to handling.

### 2.16. Statistical Analysis

The results are expressed as mean ± standard deviation (SD). Student's *t*-test used for significance analysis. *p* < 0.05 is considered as statistically significant.

## 3. Results

### 3.1. Optimizing Method for the Preparation of Mahanine-Enriched Fraction (MEF) with High Yield

Mahanine has been extensively studied for its antiproliferative activity in different cancers both *in vitro* and *in vivo* [6, 10]. Accordingly, we wanted to prepare a fraction with the highest amount of mahanine with better yield, so that this may be easy to commercialize and possibly made available as a phytopharmaceutical for the management of cancer. Accordingly, we have prepared MEF by using different solvents, namely, ethanol, water, and ethyl acetate, to prepare a fraction with the highest amount of mahanine with better yield.

**Figure 1:**
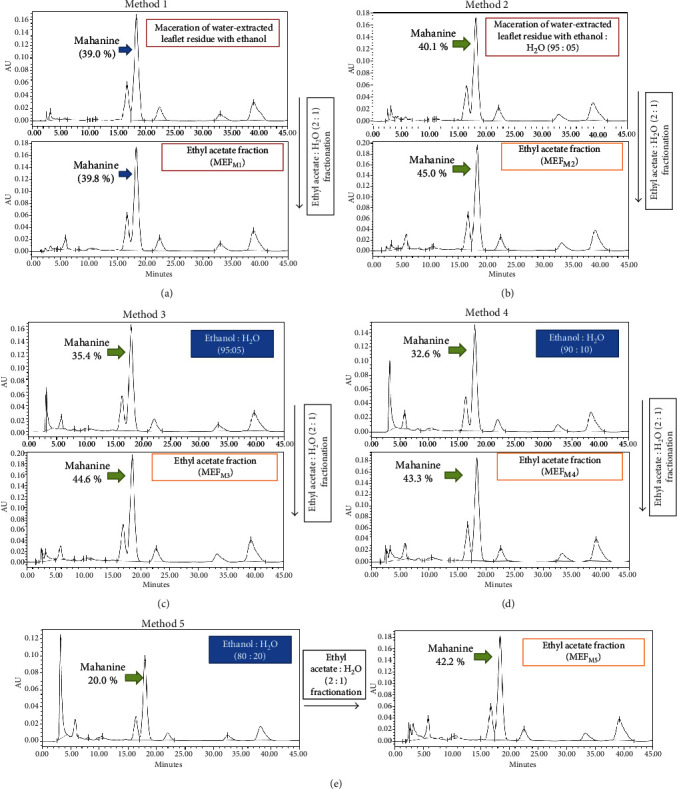
Optimization of the method for the preparation of a mahanine-enriched fraction (MEF) by HPLC. (a) HPLC profiles of the ethanolic extract of the water-extracted *M. koenigii* leaflet residue and ethyl acetate fraction. Arrow indicates the area of the peak from which percent of mahanine was calculated. (b) HPLC profiles of the ethanol : water (95 : 5) extract of the water-extracted *M. koenigii* leaflet residue and ethyl acetate fraction. (c–e) HPLC profiles of Methods 3-5 for the preparation of MEF_M3-M5_ involved in the initial maceration of leaflets with ethanol : water in different ratios. This step is followed by fractionation with ethyl acetate. The mahanine peaks in both steps are indicated by arrows. The HPLC profile of MEF has been reported earlier [14].

Initially, leaflets were infused with water and mahanine content was checked by HPLC (Method 1). No mahanine was found in this aqueous fraction and was thus discarded (Figure 1(a), Table 2). The remaining residue was macerated with ethanol and fractioned using ethyl acetate and water to further remove water-soluble matter. The yield and mahanine percent of this final product (MEF_M1_) were 3.92 g and 39.8%, respectively, as determined by HPLC.Additionally, for comparision, we have repeated our previously reported method [14] with these leaflets (50.0 g) and obtained MEF with 8.0 % yiled (4.0 g) and with 38.0 % mahanine (data not shown), which is less than MEF_M1_.

For Method 2, leaflets were similarly infused with water and the remaining residue was first extracted with ethanol : water (95 : 5). HPLC analysis of this extract showed a mahanine content of 40.1%. Next, water-soluble molecules from this extract were removed by fractionation using ethyl acetate and water. HPLC analysis of this final product (MEF_M2_) revealed 45.0% mahanine indicating ~4.9% enrichment (Figure 1(b)).

In Methods 3, 4, and 5, we used ethanol and water at ratios of 95 : 05, 90 : 10, and 80 : 20, respectively, for maceration and the extracts showed 35.4%, 32.6%, and 20.0% of mahanine, respectively (Figures 1(c)–(e)). This step was followed by fractionation with ethyl acetate to prepare the mahanine-enriched fractions MEF_M3_, MEF_M4_, and MEF_M5_, respectively.

The percentage of mahanine in MEF_M3_, MEF_M4_, and MEF_M5_ were 44.6, 43.3%, and 42.2%, respectively. The amount of MEF_M1_, MEF_M2_, MEF_M3_, MEF_M4_, and MEF_M5_ from 100 grams of dry leaflets (yield %) were found to be 7.8%, 7.3%, 6.5%, 6.5%, and 5.8%, respectively (Table 2). Additionally, both MEF_M2_ and MEF_M3_ exhibited the highest percent of mahanine (~45.0%). However, MEF_M2_ showed a better yield with a high amount of mahanine, concluding that 5% of the water in ethanol is the best ratio for enrichment. Therefore, given all these points, Method 2 may be the best method which could lead to a decrease of the cost of products and could be commercially viable.

#### 3.1.1. Characterization of MEF

In general, the qualities of five different MEFs were determined mainly based on the presence of the highest amount of mahanine, the most biologically active carbazole alkaloid by HPLC (Figure 1). Additionally, the HPLC fingerprints were compared for the presence of two more carbazole alkaloids, namely, mahanimbine and koenimbine which were characterized earlier [14]. The mahanine peak in MEF_M2_ was also confirmed by mass spectrometry.

### 3.2. MEF_M2_ Exhibited Antiproliferative Activity against Ovarian and Breast Cancer Cells

MEF_M2_ (0-40 *μ*g/mL) exhibited antiproliferative activity in a concentration-dependent manner against two representative breast cancer cell lines (MCF7 and 4T1) with different mutation status (Table 1). IC50 values were in the range of ~14.4-16.2 *μ*g/mL.

### 3.3. MEF_M2_ Induced Apoptosis in Ovarian Cancer Cells

**Figure 2:**
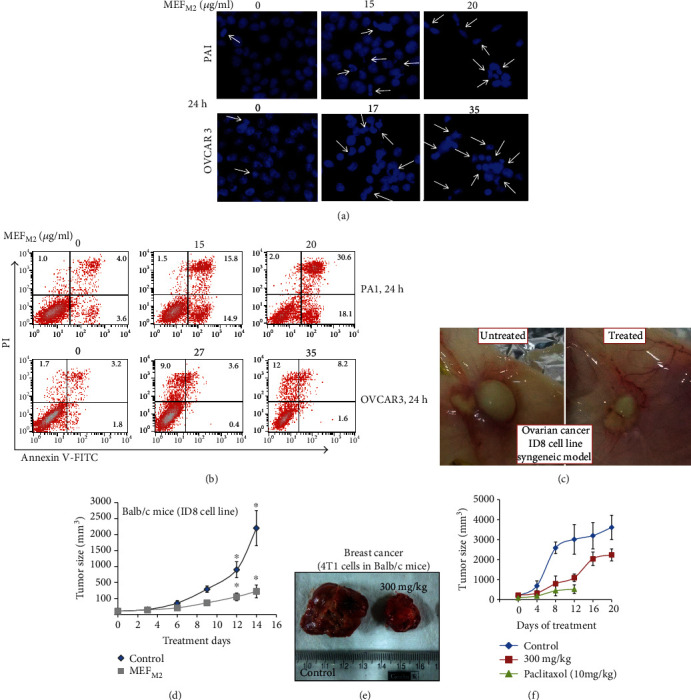
MEF_M2_ induced apoptosis in cancer cells and reduced tumor mass in syngeneic cancer mouse models. (a) Ovarian cancer cell lines were treated with MEF_M2_ at different concentrations and stained with DAPI. The images were captured under a florescence microscope. Arrows indicate the condensed and fragmented nucleus. (b) MEF_M2_-treated ovarian cancer cells were stained with annexin V/PI and analyzed by FACS. (c) A representative image of the ID8 tumors from the control and the treated mouse. (d) Graph showing the tumor size variation between MEF_M2_ treated (300 mg/kg BW/day) and vehicle control in ovarian cancer-bearing Balb/c mice. (a–d) Similar experiments were previously done with pure mahanine [10]; however, here we repeated the experiment with MEF_M2_ for comparison. (e) A representative image of the 4T1 tumors from the control and the treated mouse. (f) Graph showing the tumor size variation between MEF_M2_ treated (300 mg/kg BW/day), vehicle control, and positive control (paclitaxel) in breast cancer-bearing Balb/c mice. Error bars in all graphs represent the mean ± SD. There is a significant difference (*p* < 0.05) between control and treated groups in their tumor sizes and body weights.

DAPI staining of 24 h treated ovarian cancer cells showed a fragmented/condensed nuclei suggesting MEF_M2_-induced apoptosis (Figure 2(a)). Moreover, MEF_M2_ (24 h)-treated PA1 and OVCAR3 cells showed increased annexin-V/PI positivity from ~8.6 to ~50.7% and ~6.7 to ~21.8%, respectively, confirming the programmed cell death (Figure 2(b)).

### 3.4. MEF_M2_ Exhibited *In Vitro* and *In Vivo* Efficacy against Breast Cancer Cells and a Syngeneic Mouse Model

To check the *in vivo* efficacy of MEF_M2_, we generated a syngeneic mice model of ovarian cancer. A dose of 300 mg/kg body weight/day MEF_M2_ for fourteen days inhibited the tumor size as well as growth rate of both cancers (Figures 2(c) and 2(d)).

Additionally, we also tested MEF_M2_ in a breast cancer model to understand its application in a wide range of cancers. We have used three different doses from 300 to 900 mg/kg BW to determine the ED50 in the same experiment. However, we found an ~2-fold reduction in tumor size at the end of 20 days of feeding with MEF_M2_ even at a dose of 300 mg/kg BW (Figures 2(e) and 2(f)). In parallel, paclitaxel, a well-known first-line anticancer drug, was used as a positive control. Interestingly, all paclitaxel-treated animals died within 12 days compared to the MEF_M2_-treated mice, indicating the improved potential of MEF_M2_ (Figure 2(f)). Therefore, 300 mg/kg BW may be considered as ED50. Similar trends were observed in cancer-bearing mice from the 600 and 900 mg/kg BW groups. Additionally, we observed a greater difference in tumor size between the treated and control groups in breast cancer mice on the 12th day of treatment. Moreover, we found that there is no weight loss during and after the treatment period from doses of up to 900 mg/kg BW, again proving the safety of MEF_M2_ (Figure S1, Table S1).

### 3.5. MEF_M2_ Is Stable at Different Temperatures (-20 to 40°C)

For the development of any phytopharmaceutical, stability information of the product is essential as it contributes to the efficacy of a drug or its dosage form. Accordingly, MEF_M2_, kept at different temperatures (40, 25, 4 and -20°C), light conditions, and storage container material (glass or polypropylene) for 90 days showed no significant variation in IC50 against PA1 compared to freshly prepared MEF_M2_ indicating its long-term stability under all these conditions (Table 3).

### 3.6. MEF_M2_ is Stable at a Wide Range of pH (1-10) without Losing Its Biological Activity

Any oral drug that enters into the human body is exposed to different pH conditions, For example, the stomach is highly acidic with a pH of 1.5–4.0 and the lower intestine is in basic pH of 7–8.5 which can significantly affect bioavailability. Even after absorption, the drug will be exposed to neutral pH in the blood (7.2–7.4). Therefore, analyzing the pH stability of any therapeutic agent is necessary.

**Figure 3:**
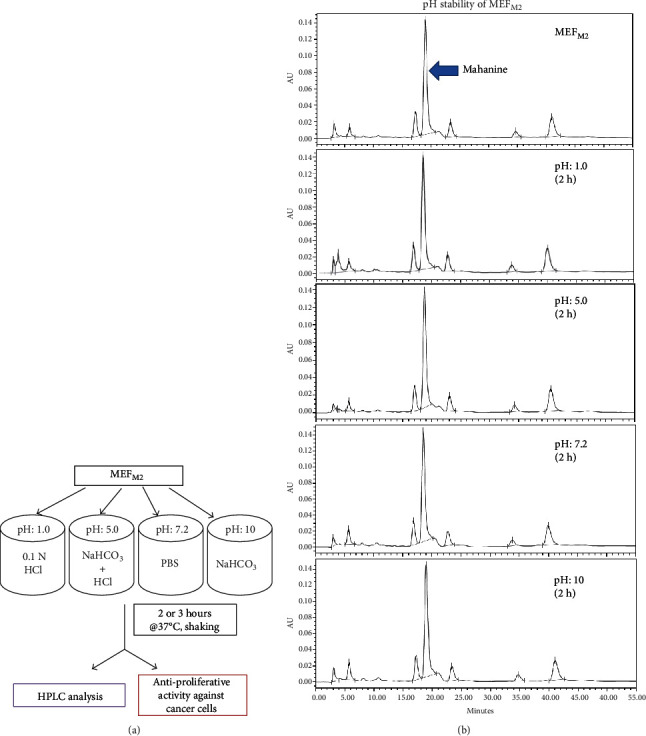
MEF_M2_ is stable at a wide range of pH. (a) Schematic image showing the steps involved in MEF_M2_ pH stability for 2-3 h analysis. (b) A representative HPLC profile of freshly prepared MEF_M2_ and the same exposure to different pH conditions for 2 h. The mahanine peak is indicated with an arrow.

Here, we have checked the stability of MEF_M2_ at different pH ranging from 1 to 10 (Figure 3(a)). As the average meantime of any oral drug in the stomach is not more than three hours, we have checked pH stability for 2-3 hours through *in vitro* MTT assay and by HPLC to detect the change in mahanine quantity (Figure 3(b)). The relative percent of mahanine, defined as total mahanine in freshly prepared MEF_M2_, was considered as 100% (Table 4). The percent of mahanine remains unchanged when MEF_M2_ was exposed to neutral or basic pH. There was a slight decrease in mahanine (0.8-8.6%) at acidic pH after 2-3 h. However, there was no significant variation in IC50 against PA1 compared to freshly prepared MEF_M2_ indicating its stability at a wide range of pH (1-10) for at least 3 hours without losing its biological activity.

### 3.7. Comparative Pharmacokinetics of Mahanine between MEF_M2_ and Mahanine in Fed Rats

**Figure 4:**
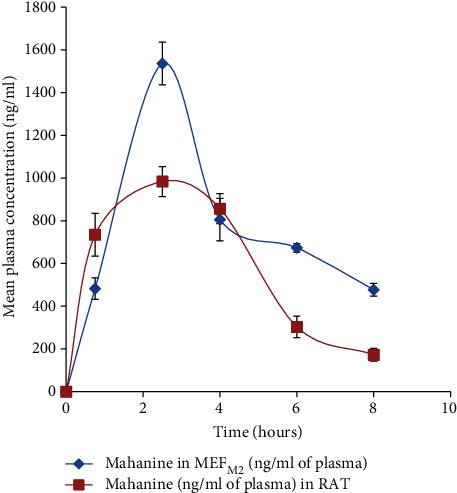
Time vs. concentration curve (area under the curve (AUC)) of mahanine in MEF_M2_- and mahanine-fed rat plasma. Error bars in all graphs represent the mean ± SD. There is a significant difference (*p* < 0.05) between the two groups.

The pharmacokinetic study of MEF_M2_ was based on the estimation of the most stable marker in plasma [18]. Mahanine was found to be the most stable, major marker and was therefore chosen for pharmacokinetics studies. A comparative pharmacokinetics study was carried out concerning mahanine following single-dose oral administration of MEF_M2_ (300 mg/kg BW) and pure mahanine (an equivalent of 300 mg/kg MEF_M2_) separately in Wistar rats. Mean plasma concentration versus the time profile for mahanine is represented in Figure 4. Maximum plasma concentrations (*C*_max_) of 1536 and 984 ng/mL were reached within 2 h of feeding (time to reach maximum concentration (*T*_max_)) for MEF_M2_ and mahanine, respectively. Areas under the curve (AUC_0–*t*_ and AUC_0–*∞*_) were 6332 and 4823 ng·h/mL and 9984 and 5254 ng·h/mL MEF_M2_ and mahanine, respectively (Table 5). Since mahanine is not soluble in water, we cannot admit it through the intravenous route. Therefore, the relative oral bioavailability (*F* = AUC_0–*t*_ (test)/AUC_0–*t*_ (reference) × 100%) of MEF was calculated. Based on this calculation, MEF_M2_ showed ~31% more bioavailability compared to pure mahanine.

### 3.8. Single-Dose Maximum Tolerated Dose (MTD) of MEF_M2_ Is ~5000 mg/kg BW

Animals fed with 2000 mg/kg BW/day of MEF_M2_ did not cause mortality or any clinical signs of acute toxicity within 48 h and within a 14-day period. Although the locomotor activities of the mice fed with 5000 mg/kg BW/day were slightly reduced and the fur became rough after 48 h, nevertheless, this group of animals recovered quickly and no mortality was found up to 14 days.

**Figure 5:**
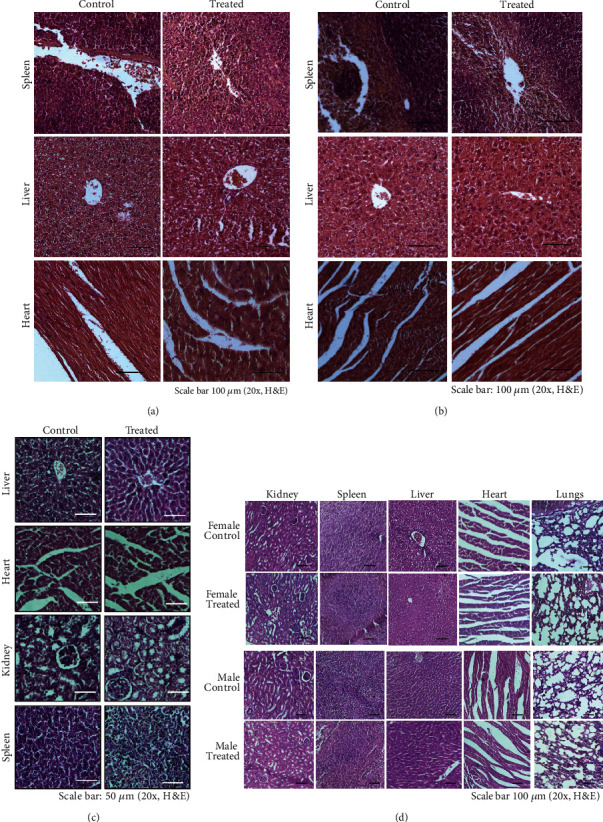
Histopathology of different organs from mice of acute, subchronic, and chronic toxicity studies. (a) Organs from control and 5000 mg/kg MEF_M2_-fed mice were isolated. These are sectioned and stained with Haemotoxylin and Eosin (H&E) for histopathological observation as discussed in Methods. (b) Histopathology of different organs collected from 14-day-fed mice. (c) Histopathology of different organs collected from 90-day-fed mice (sub-chronic toxicity study). (d) 180-day-fed mice (chronic toxicity study) were similarly stained for histopathology study. No significant changes or damages were observed in tissues of MEF_M2_-fed mice compared to the control groups. All are representative images from a single mouse from each group. Magnification (20x) and scale bar are shown in all the images.

No weight loss among mice from both groups was observed (Table 6). No abnormality in the organs as observed by histopathology studies and no significant change in serum biochemistry were observed as compared to the animals fed with vehicle. A representative image of H&E-stained organs and the serum chemistry of mice fed with 5000 mg/kg BW are presented in Figure 5(a) and Table 7, respectively.

### 3.9. Repeated-Dose MTD of MEF_M2_is >1000 mg/kg BW/Day

A daily dose of MEF_M2_ from 300 to 1500 mg/kg BW/day for 14 days in individual groups of mice showed no change in general behavior like breathing, fur texture, water consumption, and impairment in food intake, and there was no loss of body weight in doses of up to 1000 mg/kg BW/day during the study period (Table 8). However in the 1500 mg/kg BW/day group, the mice were slightly inactive, indicating that the repeated-dose MTD is between 1000 and 1500 mg/kg BW/day.

### 3.10. MEF_M2_ Exhibited No Apparent Acute, Subacute, Subchronic, and Chronic Oral Toxicity

Daily oral administration of MEF_M2_ (300 mg/kg BW) for 14, 28, 90, and 180 consecutive days did not induce any noticeable symptom of toxicity in mice. No lethality was recorded during the study period. No differences in general behavior, food, and water consumption were observed between the treated and untreated groups in all these studies.

**Figure 6:**
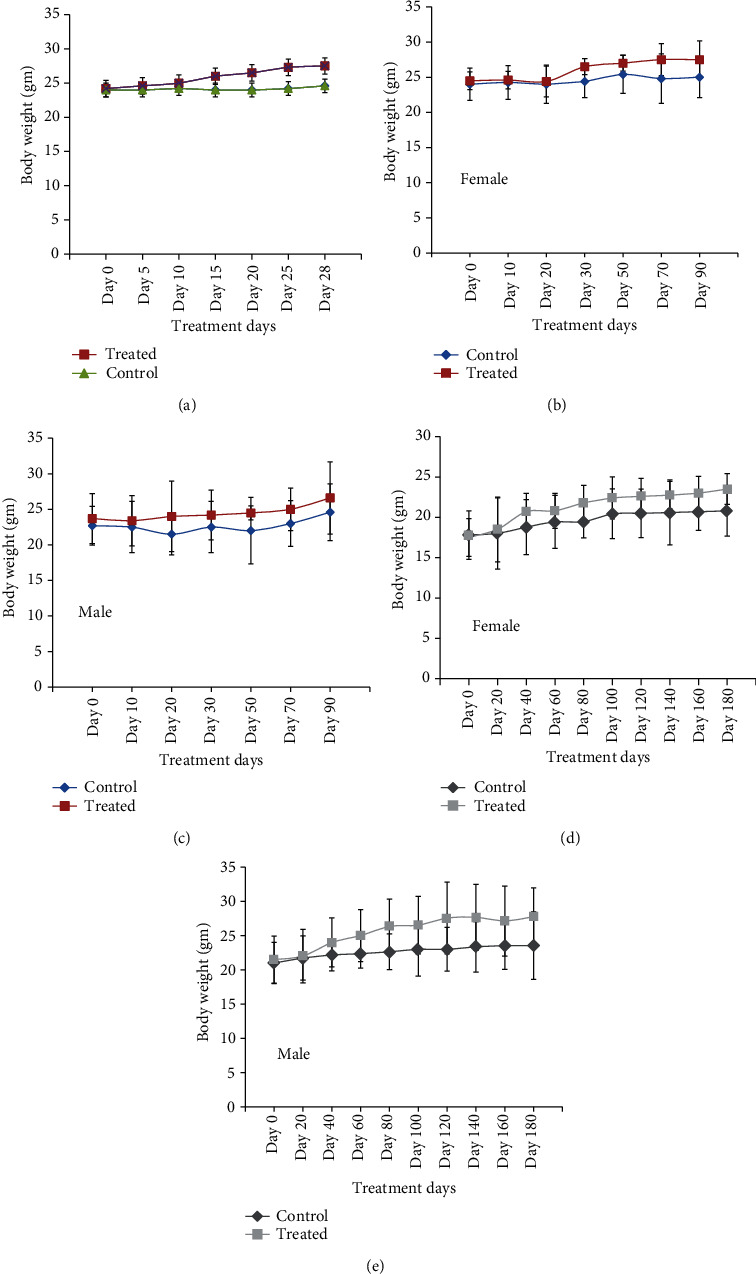
Body weights of control and MEF_M2_-fed mice for 28, 90, and 180 days. (a) MEF_M2_ was fed to female mice for 28 days, and body weights were monitored during the treatment period and presented in a graph. (b and c) Body weights of MEF_M2_-fed male and female mice for 90 days and compared to vehicle-fed mice (control). (d–e) Body weights of 180-day-fed (MEF_M2_/vehicle) male and female mice are plotted for comparison. Error bars in all graphs represent the mean ± SD. There are no significant (*p* > 0.05) differences between the control and treated mice body weights in all groups.

MEF_M2_ slightly increased the body weights of mice in the subacute toxicity study (Figure 6(a); Table S2). Similar results were observed in subchronic (Figures 6(b) and 6(c); Table S3) and chronic toxicity studies both in male and female treated animals (Figures 6(d) and 6(e); Table S4).

Additionally, the serum biochemistry of all experimental animals at 14 (Table 9), 28 (Tables 10), 90 (Tables 11), and 180 (Tables 12) days showed no significant changes between treated and untreated groups. However, in the subchronic study, female mice showed a slight increase in liver enzymes (ALT/ALP) and triglycerides with a slight decrease in GGT with no change in LDH. In contrast, treated male mice showed a slight decrease of ALT and ALP and a small enhancement of LDH, while the level of triglycerides remains unchanged. Additionally, in the chronic study, there was a slight decrease (insignificant) in triglycerides in female treated mice with unaltered ALT, ALP, GGT, and LDH. In male mice, there was a minor decrease in ALT levels and a slight increase in GGT and total cholesterol with unchanged levels of triglycerides.

Moreover, the weights of major organs including the heart, spleen, liver, lungs, and kidneys were not reduced in the MEF-fed group of mice in both sexes compared to the vehicle-fed animals in subchronic (Table 13) and chronic (Table 14) toxicity studies.

Additionally, the hematological parameters of treated mice of both sexes showed no significant changes compared to untreated mice in the subchronic toxicity study (90 days). However, there was a slight increase in ESR level (0.2- to 0.4-fold; Table 15).

Furthermore, the histopathology of the kidney, spleen, liver, heart, and lung from a representative mouse of each group in acute (Figure 5(b)), subchronic (90 days; Figure 5(c)), and chronic (180 days) toxicity studies (Figure 5(d)) showed no visible histomorphological damages. Taken together, based on these detailed toxicity studies, MEF_M2_ is found to be nontoxic to mice even after feeding for 180 days.

## 4. Discussion

The main achievement of this study is the optimization of a method for the preparation of a mahanine-enriched fraction (MEF) having the highest percent of mahanine and a good yield with all the necessary preclinical data including efficacy against syngeneic cancer models, long-term safety, stability, and pharmacokinetics, which are needed for submission to the regulatory body for clinical trial approval.

Earlier, our group had isolated and extensively studied a carbazole alkaloid, mahanine, from the leaflets of a medicinal plant *M. koenigii* [6, 10]. However, purification of mahanine from leaflets involves many steps resulting in a minute amount of pure molecules. Moreover, the total synthesis of mahanine also involved many steps with a low yield [19]. Unfortunately, the synthesized molecule is a mixture of both Levo and Dextro isomers, whereas Levo is available in this plant.

Here, we could successfully standardized a method (Method 2, MEF_M2_) to prepare MEF in which we acquire the highest mahanine content as well as the maximum total yield simply through the maceration of leaflets with ethanol : water in a 95 : 5 composition by comparing five different methods using only three solvents with different compositions of ethanol, water, and ethyl acetate. As mahanine is insoluble in water, we have incorporated simple steps to remove the total water-soluble portion from these leaflets to improve the enrichment. Accordingly, water infusion before maceration with ethanol followed by the fractionation of the obtained extract with ethyl acetate gave the best results. Although Method 1 and our previously reported method [14] showed the highest yield (7.8 and 8.0 respectively), we have selected Method 2 because of its high percent and maximum amount of mahanine. A high content of mahanine in MEF_M2_ was further supported by *in vitro* activity against both ovarian and breast cancer cells. Earlier, we have reported the anticancer activity of MEF both in ovarian and lung cancer xenograft models [14]. Here, we showed the excellent *in vivo* efficacy of MEF_M2_ in the ovarian and breast cancer syngeneic models at a dose of 300 mg/kg BW. Moreover, in both ovarian and breast cancer models, treated mice showed a significant difference in tumor size after 14 days of MEF_M2_ feeding. However, we continued feeding up to 20 days in the breast cancer model to check the survivability and any body weight loss. Interestingly, these MEF_M2_-treated breast cancer-bearing mice did not show any significant body weight loss even up to 900 mg/kg for 20 days, indicating no apparent toxicity.

One of the major problems in cancer is lower survivability [20]. Paclitaxel is an important drug used regularly in breast cancer [21]; however, it is unable to increase the survivability. Here, interestingly, we found that MEF-treated cancer-bearing mice even at the lower dose of 300 mg/kg BW survived for a longer time compared to the paclitaxel-treated group indicating the potentiality of MEF for the management of this cancer.

The stability of a drug ensures its quality, safety, efficacy, and shelf life [22]. Therefore, detailed information on the stability of any pharmaceutical product is a prerequisite for its acceptance and approval by regulatory authorities [23]. The stability checking depends on the exposure of herbal products to different environmental factors, like temperature, humidity, light, and the material of the storage container, which determine their shelf lives [22]. Previously, we have demonstrated that the biological activity of MEF against cancer cells is directly proportional to the mahanine content present in the MEF [14]. Our goal is to check the biological activity of MEF after incubating at different temperatures. Therefore, we aimed to check the biological activity of MEF through MTT, which is more crucial. Here, we found that MEF did not lose biological activity against cancer cells when it was exposed to 40°C in glass or polypropylene containers for >3 months in both light and dark conditions assuring its high stability.

Orally administered drugs are usually exposed to different pH conditions of the intestinal tract ranging from a pH of 1.0 to 10 [24]. Furthermore, pH also influences the chemical stability and pharmacokinetics of phytochemicals [25]. Therefore, the pH stability of a drug is essential for its ultimate biological activity. Here, we found that MEF did not lose its bioactivity against cancer cells even after exposure to a wide range of pH (1-10). Additionally, we have shown that at higher acidic conditions of around pH 1.0, mahanine quantity was reduced by ~9.0%; however, there was no loss in its overall biological activity, suggesting also the same effect on the activity of its metabolites. All these observations suggest that MEF has good pH stability. Accordingly, MEF could be prepared as oral dosage forms like tablets, capsule, or syrup.

Pharmacokinetics, especially oral bioavailability, decides the dose of a drug [26]. Unlike pharmaceuticals, herbal drugs are a mixture of different unknown compounds. Therefore, it is a great challenge to check the pharmacokinetics of herbal products [17]. However, using a major biologically active marker compound like mahanine can be utilized for this purpose. Here, we found that MEF showed the good bioavailability of mahanine suggesting that mahanine present in MEF is easily absorbable through the oral route. Interestingly, we observed that MEF showed higher *C*_max_ compared to the *C*_max_ of pure mahanine. This may be due to the presence of other molecules present in MEF_M2_ which might compete with mahanine for the metabolism in the liver that leads to increased plasma concentration.

The major problem with phytochemicals is the lack of long-term safety evaluation data [2]. Therefore, it is essential to collect detailed information on the toxicity of MEF in normal animals before entering into clinical trials. As a part of the toxicity study, we analyzed blood parameters to check for any possible toxicity that affects liver/kidney functions. Additionally, we checked the toxicity of tissues in MEF_M2_-fed normal mice by H&E staining. Here, we observed that MEF did not induce death even up to 5000 mg/kg BW/day, which is ~17-fold higher than the ED50 dose (300 mg/kg BW/day) indicating its high therapeutic index. In the acute repeated-dose study, we observed no clinical signs of toxicity at 1000 mg/kg BW/day for fourteen days, indicating its higher MTD. Furthermore, feeding of MEF for 14, 28, 90, and 180 days did not show any abnormal weight loss and no drastic changes in serum chemistry in mice. All these extensive studies indicate the safe use of MEF for up to 180 days.

Taken together, such a mahanine-enriched fraction with an HPLC fingerprint for quality assessment, good pH and temperature stability, *in vitro* and *in vivo* efficacy, pharmacokinetics, and long-term toxicity studies would be helpful for the preparation of the best phytopharmaceutical for the management of cancer from this edible plant.

## 5. Conclusions

The preparation of a mahanine-enriched fraction (MEF) was optimized for a better yield with a high amount of mahanine. MEF_M2_ exhibited reduced cell viability in both ovarian and breast cancer cell lines. Additionally, we have demonstrated that MEF_M2_ induced apoptosis in a representative ovarian cancer. It also reduced tumor growth in ovarian and breast cancer syngeneic models at 300 mg/kg, which is comparatively safer than paclitaxel, a known clinically used drug. Most importantly, the orally fed MEF_M2_ animals did not show any acute toxicity in up to ~5000 mg/kg BW single-dose and >1000 mg/kg BW repeated dose for 14 days. Furthermore, at ED50 (300 mg/kg), it did not show any subacute (28 days), subchronic (90 days), and chronic (180 days) toxicity. MEF is stable in both plastic and glass vials up to 40°C for ≥3 months. A wide range of pH (1-10), stability, and good bioavailability of MEF allows its safe and effective oral administration.

Taken together, all these preclinical data of MEF_M2_ will act as a reference material for submission to regulatory bodies for clinical trial approval.

## Figures and Tables

**Table 1:** Antiproliferative activity of MEF_M2_ in ovarian and breast cancer cell lines.

Cancer type	Cell lines	Mutation/drug resistant	Culturing media + 10% FBS	MEF_M2_IC50 (*μ*g/mL)
Ovarian (human)	PA1	NRAS mutation, anoikis resistant	MEM	14.5 ± 0.15
	OVCAR3	TP53 mutation, drug resistant	IMDM	26.2 ± 0.2
Ovarian (mouse)	ID8	TP53, BRCA1, and BRCA2 wild type	RPMI-1640	17.1 ± 0.02
Breast (human)	MCF7	ESR1 mutant	MEM	14.4 ± 0.22
Breast (mouse)	4T1	P53 null mutant	RPMI-1640	16.2 ± 0.22

In vitro activity of MEF_M2_ in ovarian and breast cancer cell lines was presented. This data was derived from three individual experiments and the mean ± SD was indicated for IC50 values. Anti-proliferative activity of MEF [14] and its biomarker, mahanine [10], in PA1 and OVCAR3 cell lines were previously reported by our group, however we have repeated here with MEF_M2_ for comparison.

**Table 2:** Comparison of five different methods for the preparation of MEF from *M. koenigii* leaflets.

Steps	Method 1	Method 2	Method 3	Method 4	Method 5
Dry leaflets (starting material)	50 grams	50 grams	50 grams	50 grams	50 grams
Infusion with water (50°C)	Water fraction was discarded	Water fraction was discarded	—	—	—
Maceration with solvent for (72 h)	Ethanol (water-extracted leaflet residue)	Ethanol : H_2_O (95 : 05) (water-extracted leaflet residue)	Ethanol : H_2_O (95 : 05)	Ethanol : H_2_O (90 : 10)	Ethanol : H_2_O (80 : 20)
Weight of extract (grams) (yield %)	4.12 (8.24)	4.00 (8.0)	4.9 (9.8)	6.0 (12)	7.86 (15.72)
Mahanine (%) in extract by HPLC	39.0	40.1	35.4	32.6	20.0
Fractionation with ethyl acetate : water (3 : 1) Weight of fraction (final product in grams)	3.92	3.66	3.24	3.25	2.91
Yield (%) (amount of fraction/100 grams of dry leaflets)	7.8	7.3	6.5	6.5	5.8
Comparison of mahanine (%) in extract and fraction by HPLC	Enriched to 39.8	Enriched to 45.0	Enriched to 44.6	Enriched to 43.3	Enriched to 42.2
Total amount of mahanine in final product (mg)	1560	1640	1450	1410	1230

Five different methods showing the enrichment of the mahanine percent yield of MEF. This experiment was repeated at least thrice to confirm the results.

**Table 3:** Stability of MEF_M2_ at different temperatures, light and storage containers.

S. No.	MEF_M2_ stored for 90 days at temperature (°C)	Relative humidity (%)	Container material	Storing conditions	IC50 values (*μ*g/mL) in PA1 cell line
1	40 ± 2	75 ± 5	Glass	Dark	14.8 ± 0.25
2	25 ± 2	60 ± 5	Glass	Dark	14.3 ± 0.15
3	5 ± 3	—	Glass	Dark	14.0 ± 0.10
4	−20 ± 5	—	Glass	Dark	14.2 ± 0.05
5	25 ± 2	60 ± 5	Glass	Light	14.3 ± 0.30
6	25 ± 2	60 ± 5	Polypropylene	Light	13.9 ± 0.12
7	5 ± 3	—	Polypropylene	Dark	14.24 ± 0.27
8	−20 ± 5	—	Polypropylene	Dark	14.25 ± 0.15
9	Freshly prepared MEF_M2_	—	—	—	14.0 ± 0.25

This data was derived from three individual experiments and the mean ± SD was indicated. PA1 is used as a representative cell line for these studies.

**Table 4:** pH stability of MEF_M2_.

	Exposure of MEF_M2_ at different pH	Relative % of mahanine after		IC50 (*μ*g/mL) of MEF_M2_ after 3 h exposure against PA1 cells
		2 h	3 h	
Freshly prepared MEF_M2_	—	100	100	14.4 ± 0.3
	pH 1.0	99.2	91.4	13.5 ± 0.15
	pH 5.0	100	96	13.9 ± 0.05
	pH 7.2	100	100	14.3 ± 0.2
	pH 10	100	98.7	14.7 ± 0.35

This data was derived from three individual experiments, and the mean ± SD is indicated.

**Table 5:** Comparative pharmacokinetic parameters of MEF_M2_ and mahanine in rats.

Sample	Dose (mg/kg BW)	*C*_max_ (ng/mL)	Area under the curve (AUC)_0−*t*_ (ng·h/mL)	AUC_∞_ (ng·h/mL)	*T*(1/2) (h)	Mean residence time [MRT (h)]	*T*_max_ (h)
MEF_M2_	300 (P.O.)	1536	6332	9984	5.3	8.15	2.5
Mahanine	Equivalent to 300 (P.O.) of MEF_M2_	984	4823	5254	1.8	3.9	2

**Table 6:** Single-dose MTD study of MEF_M2_.

Single-dose (mg/kg BW) (*n* = 5)	Weight (grams)		Activity and mortality (live) after 14 days
	Days		
	0	14	
2000	20 ± 2.1	23.5 ± 0.6	Live and active
5000	21 ± 1.6	22.2 ± 1.1	Live and active

Data indicate mean ± SD. There are no significant (*p* > 0.05) differences between the control and treated mice in their body weights.

**Table 7:** Serum biochemistry of mice fed with a single dose of 5000 mg/kg.

Test name (units)	Untreated mice	Mice treated with MEF_M2_ (5000 mg/kg BW)
Urea (mg/dL)	25.6 ± 4.3	27.2 ± 2.3
Cholesterol (mg/dL)	83.5 ± 1.3	84.3 ± 1.2
SGOT (U/L)	133.5 ± 5.5	123.9 ± 10.1
Total protein (g/dL)	7.3 ± 1.1	6.6 ± 1.5
Albumin (ALB, g/dL)	3.7 ± 0.3	3.5 ± 0.05
Globulin (GLB, g/dL)	3.6 ± 0.2	3.1 ± 0.03
ALB : GLB	1.03 : 1	1.13 : 1
Creatinine (mg/dL)	0.69 ± 0.03	0.51 ± 0.25

Serum was collected after 14 days and analyzed for different biochemical parameters in comparison with untreated mice. Data indicate mean ± SD. There are no significant (*p* > 0.05) differences between the control and treated mice in their biochemical parameters.

**Table 8:** Maximum tolerated dose (MTD) of MEF_M2_.

Dose (mg/kg/day), fed for 14 days	Weight (grams) on the day of treatment	Weight (grams) after 14 days	Behavioral patterns, activity during feeding period
Control	24.5 ± 0.8	26 ± 0.6	Normal
300	24 ± 1.1	25 ± 0.4	Normal
500	25 ± 0.7	27 ± 0.3	Normal
750	22.5 ± 0.2	24 ± 0.9	Normal
1000	23 ± 0.6	23.5 ± 1.0	Normal
1500	23 ± 1.5	22 ± 1.2	Slightly inactive

Normal Balb/c mice were fed for 14 days to determine MTD. Body weights are represented by mean ± SD. There are no significant (*p* > 0.05) differences between the control and treated mice.

**Table 9:** Serum biochemistry of mice fed with MEF_M2_ for 14 days.

Test (units)	Control	Treated
Urea (mg/dL)	25.4 ± 1.3	27.3 ± 2.3
Creatinine (mg/dL)	0.47 ± 0.06	0.57 ± 0.05
Total cholesterol (mg/dL)	81.6 ± 3.4	86.2 ± 2.5
SGOT (U/L)	127.4 ± 4.1	131.5 ± 3.9
Total protein (g/dL)	6.2 ± 0.5	5.8 ± 0.8
Albumin (ALB, g/dL)	3.2 ± 0.2	2.7 ± 0.5
Globulin (GLB, g/dL)	3.0 ± 0.3	2.8 ± 0.3
ALB : GLB	1.07 ± 0.05 : 1	0.89 ± 0.11 : 1

Control mice fed with vehicle. SGOT: serum glutamic oxaloacetic transaminase. Data indicate mean ± SD. There are no significant (*p* > 0.05) differences between the control and treated mice in their biochemical parameters.

**Table 10:** Serum biochemistry of the subacute toxicity study group of mice.

Test (units)	Control	Treated	Fold change
Urea (mg/dL)	39.2 ± 7.1	40.5 ± 0.7	1.03
BUN (mg/dL)	18.46 ± 3.4	19.05 ± 0.35	1.03
Creatinine (mg/dL)	0.6 ± 0.09	0.61 ± 0.1	1.1
ALT (U/L)	79.5 ± 12.3	86.6 ± 30.9	1.09
ALP (U/L)	207.6 ± 36.1	194 ± 21.2	0.94
GGT (U/L)	1 ± 0.15	0.9 ± 0.1	0.9
Bilirubin (mg/dL)	0.4 ± 0.07	0.46 ± 0.11	1.15
Total cholesterol (mg/dL)	136.8 ± 12	161 ± 14	1.16
Triglycerides (mg/dL)	224.5 ± 27.5	270.5 ± 30.5	1.2
SGOT (U/L)	201 ± 40.2	157 ± 45.6	0.78
Total protein (g/dL)	5.94 ± 0.32	6.9 ± 0.02	1.16
Albumin (ALB, g/dL)	3.41 ± 0.19	4.19 ± 0.08	1.23
Globulin (GLB, g/dL)	2.53 ± 0.39	2.68 ± 0.1	1.06
ALB : GLB	1.24 ± 0.13	1.56 ± 0.09	1.25
LDH (U/L)	3228.8 ± 633.8	2834 ± 293	0.88

Fold change (control/treated value) is the relative value where the control value is considered as 1.0 and the treated value is compared with it. Data indicate mean ± SD. There are no significant (*p* > 0.05) differences between the control and treated mice in their biochemical parameters.

**Table 11:** Serum biochemistry of mice in the subchronic toxicity study.

Test (units)	Female		Fold change	Male		Fold change
	Control	Treated		Control	Treated	
Urea (mg/dL)	43.4 ± 3.28	47.3 ± 6.2	1.09	41 ± 6.2	47.1 ± 6.9	1.15
BUN (mg/dL)	20.46 ± 1.56	22.3 ± 2.9	1.09	19.3 ± 2.68	22.2 ± 3.28	1.15
Creatinine (mg/dL)	0.306 ± 0.06	0.34 ± 0.09	1.1	0.26 ± 0.11	0.28 ± 0.03	1.08
ALT (U/L)	54.5 ± 30.3	69.45 ± 25.4	1.3	76 ± 2.82	66.3 ± 18.5	0.87
ALP (U/L)	80.4 ± 21.9	122.6 ± 31.3	1.5	162 ± 35.4	97 ± 22.3	0.6
GGT (U/L)	3.0 ± 1.4	2.25 ± 1.25	0.75	3.0 ± 0	2.0 ± 1.4	0.7
Bilirubin (mg/dL)	0.42 ± 0.08	0.41 ± 0.09	0.98	0.5 ± 0.1	0.57 ± 0.12	1.14
Total cholesterol (mg/dL)	95.2 ± 17.16	97.9 ± 13.3	1.03	109.7 ± 20.4	101.8 ± 13.8	0.93
Triglycerides (mg/dL)	85 ± 22.8	118.2 ± 37	1.4	129 ± 5.6	136.3 ± 39.7	1.06
SGOT (U/L)	206.5 ± 60.3	185.3 ± 41.3	0.9	217 ± 99.6	172 ± 31.8	0.8
Total protein (g/dL)	6.3 ± 0.56	6.3 ± 0.43	1	6.57 ± 0.32	6.07 ± 0.36	0.92
Albumin (ALB, g/dL)	3.36 ± 0.08	3.37 ± 0.1	1	3.33 ± 0.06	3.1 ± 0.08	0.93
Globulin (GLB, g/dL)	2.94 ± 0.08	2.94 ± 0.38	1	3.24 ± 0.06	2.96 ± 0.39	0.91
ALB : GLB	1.17 ± 0.5	1.17 ± 0.17	1	1.033 ± 0.09	1.06 ± 0.17	1
LDH (U/L)	2121 ± 204	2064 ± 323	0.97	1644 ± 158	2084 ± 388	1.3

Data indicate mean ± SD. There are no significant (*p* > 0.05) differences between the control and treated mice in their tested biochemical parameters.

**Table 12:** Serum biochemistry of treated/control mice in the chronic toxicity study.

Test (units)	Female		Fold change	Male		Fold change
	Control	Treated		Control	Treated	
Urea (mg/dL)	55 ± 8.7	50.5 ± 10.7	0.92	59.3 ± 13.4	62.5 ± 16.8	1.05
BUN (mg/dL)	26.20 ± 4.2	23.8 ± 5.1	0.91	28.07 ± 6.3	29.45 ± 7.9	1.05
Creatinine (mg/dL)	0.62 ± 0.06	0.59 ± 0.1	0.93	0.62 ± 0.14	0.64 ± 0.08	1.04
ALT (U/L)	97.83 ± 18.8	82.75 ± 30.8	0.85	85.4 ± 27.2	62.75 ± 13.5	0.73
ALP (U/L)	182 ± 33.12	171 ± 38.0	0.94	163.2 ± 34.4	142.5 ± 28.7	0.87
GGT (U/L)	2.86 ± 1.34	2.3 ± 0.58	0.82	3.5 ± 1.43	4.5 ± 2.07	1.28
Bilirubin (mg/dL)	0.575 ± 0.138	0.53 ± 0.125	0.91	0.53 ± 0.09	0.6 ± 0.16	1.15
Total cholesterol (mg/dL)	90.25 ± 8.1	92 ± 20.2	1.02	116.4 ± 35.2	142 ± 18.6	1.22
Triglycerides (mg/dL)	126.5 ± 39.5	86.25 ± 23.2	0.68	119.9 ± 30.9	104.7 ± 17.9	0.87
SGOT (U/L)	244.5 ± 34.2	245.5 ± 82.2	1.0	180.1 ± 60.1	212.75 ± 62.9	1.18
Total protein (g/dL)	6.4 ± 0.43	6.02 ± 0.9	0.94	6.81 ± 0.5	6.66 ± 0.54	0.98
Albumin (ALB, g/dL)	3.8 ± 0.16	3.6 ± 0.59	0.95	3.87 ± 0.35	3.82 ± 0.34	0.99
Globulin (GLB, g/dL)	2.62 ± 0.33	2.43 ± 0.32	0.92	2.94 ± 0.3	2.84 ± 0.29	0.97
ALB : GLB	1.45 ± 0.19	1.48 ± 0.07	1.01	1.3 ± 0.17	1.36 ± 0.13	1.02
LDH (U/L)	3122 ± 681	2912 ± 764	0.93	2704 ± 423	2654 ± 344	0.98

Data indicate mean ± SD. There are no significant (*p* > 0.05) differences between the control and treated mice in their biochemical parameters.

**Table 13:** Organ weights of mice fed with MEF_M2_ for 90 days.

Sex	Group	Heart (gm)	Spleen (gm)	Kidney (gm)	Liver (gm)
Female	Control	0.139 ± 0.018	0.158 ± 0.018	0.141 ± 0.026	1.25 ± 0.043
	Treated	0.142 ± 0.025	0.16 ± 0.04	0.148 ± 0.027	1.23 ± 0.183
	Fold change	1.0	1.02	1.05	0.98
Male	Control	0.134 ± 0.015	0.15 ± 0.013	0.172 ± 0.049	1.089 ± 0.176
	Treated	0.163 ± 0.009	0.23 ± 0.033	0.24 ± 0.086	1.364 ± 0.272
	Fold change	1.2	1.5	1.4	1.3

Data indicate mean ± SD. There are no significant (*p* > 0.05) differences between the organ weights of control and treated mice.

**Table 14:** Organ weights of mice fed with MEF_M2_ for 180 days.

Sex	Group	Heart (gm)	Liver (gm)	Spleen (gm)	Lungs (gm)	Kidney (gm)
Female	Control	0.119 ± 0.013	1.104 ± 0.125	0.136 ± 0.021	0.141 ± 0.015	0.278 ± 0.016
	Treated	0.125 ± 0.003	1.174 ± 0.144	0.158 ± 0.062	0.195 ± 0.068	0.301 ± 0.032
	Fold change	1.05	1.06	1.16	1.38	1.08
Male	Control	0.133 ± 0.023	1.156 ± 0.241	0.162 ± 0.072	0.165 ± 0.048	0.34 ± 0.057
	Treated	0.153 ± 0.02	1.32 ± 0.26	0.145 ± 0.043	0.176 ± 0.026	0.409 ± 0.086
	Fold change	1.15	1.14	0.89	1.06	1.2

Data indicate mean ± SD. There are no significant (*p* > 0.05) differences between the organ weights of control and treated mice.

**Table 15:** Hematology of MEF_M2_ fed mice for 90 days.

Parameter	Female		Fold change	Male		Fold change
	Control	Treated		Control	Treated	
Hemoglobin (g/dL)	12.75 ± 0.62	11.93 ± 0.43	0.94	12.32 ± 1.4	12.5 ± 1.9	1.01
Packed cell volume (PCV%)	38.5 ± 1.73	36 ± 1.5	0.94	37 ± 4.4	38.5 ± 5.35	1.04
Erythrocytes (×10^6^/*μ*L)	4.25 ± 0.17	4.03 ± 0.17	0.95	4.22 ± 0.4	4.27 ± 0.06	1.01
Leucocytes (×10^3^/*μ*L)	7.35 ± 1.6	7.43 ± 1.46	1.01	7.28 ± 2	7.65 ± 1.5	1.05
Neutrophil (%)	57 ± 11	60.3 ± 8.6	1.06	61.6 ± 12.5	64.1 ± 3.6	1.04
Eosinophil (%)	2 ± 0.2	2.14 ± 0.38	1.07	2.4 ± 0.5	2.17 ± 0.04	0.90
Lymphocyte (%)	43.6 ± 6.5	35.14 ± 9	0.81	33.6 ± 12.8	32.3 ± 3.4	0.96
Monocyte (%)	2.23 ± 0.5	2.42 ± 1.13	1.09	2.4 ± 0.89	2.17 ± 0.4	0.90
Erythrocyte sedimentation rate (mm/h)	12 ± 3.5	14.85 ± 4.14	1.24	10 ± 5.26	14.8 ± 4.6	1.48
Platelet (×10^6^/*μ*L)	2.96 ± 1.18	3.1 ± 1.28	1.05	2.6 ± 0.72	2.67 ± 0.85	1.03

Data indicate mean ± SD. There are no significant (*p* > 0.05) differences between the analyzed hematological parameters of control and treated mice.

## Data Availability

Data sharing does not apply to this article as no datasets were generated or analyzed during the current study.

## Abbreviations

MEF_M2_: Mahanine-enriched fraction prepared by Method 2
BW: Body weight
IC50: The concentration at which 50% of the cells are viable
MTT: 3-(4, 5-Dimethylthiazol-2-yl)-2,5-diphenyltetrazolium bromide
FBS: Fetal bovine serum
AUC: Area under the curve
AUC_0−*t*_: AUC up to the last measurable concentration
AUC_0−∞_: AUC from time 0 extrapolated to infinite time
*C*_max_: Maximum serum concentration
P.O.: Per os (oral feeding)
FACS: Fluorescence Activated Cell Sorting.

## Supplementary Materials

Supplementary Materials Table S1: body weights of vehicle-fed (control) and MEF_M2_-treated breast cancer-bearing mice. Table S2: body weights of MEF_M2_-treated/control mice in a subacute toxicity study (28 days). Table S3: body weights of MEF_M2_-treated/control mice in a subchronic toxicity study (90 days). Table S4: body weights of MEF_M2_-treated/control mice in a chronic toxicity study (180 days). Figure S1: graphical representation of body weights of control and three treated groups of breast cancer-bearing mice.
