# Supplementary material for: Preclinical Development of Mahanine-Enriched Fraction from Indian Spice Murraya koenigii for the Management of Cancer: Efficacy, Temperature/pH stability, Pharmacokinetics, Acute and Chronic Toxicity (14-180 Days) Studies
Source: Biomed Res Int. 2020 Aug 10;2020:4638132. doi: 10.1155/2020/4638132 (PMC7439207; doi:10.1155/2020/4638132)
Supplement: Supplementary Materials — Table S1: body weights of vehicle-fed (control) and MEFM2-treated breast cancer-bearing mice. Table S2: body weights of MEFM2-treated/control mice in a subacute toxicity study (28 days). Table S3: body weights of MEFM2-treated/control mice in a subchronic toxicity study (90 days). Table S4: body weights of MEFM2-treated/control mice in a chronic toxicity study (180 days). Figure S1: graphical representation of body weights of control and three treated groups of breast cancer-bearing mice. [file 4638132.f1.docx]

| **Days of treatment** | **1** | **4** | **7** | **10** | **13** | **16** | **20** |
| --- | --- | --- | --- | --- | --- | --- | --- |
| **Control** | 28.3 ± 2.3 | 26.3 ± 2.0 | 24.8 ± 2.6 | 23.2 ± 1.7 | 23.2 ± 2.6 | 22 ± 4.0 | 21.4 ± 5.1 |
| **300 mg/kg** | 24.5 ± 3.0 | 23.5 ± 3.4 | 23.25 ± 3.3 | 24.3 ± 4.2 | 25 ± 3.6 | 27.5 ± 3.0 | 27.7 ± 2.8 |
| **600 mg/kg** | 29.8 ± 2.7 | 27.2 ± 3.7 | 24.5 ± 2.6 | 25 ± 3.6 | 25.2 ± 2.7 | 26.6 ± 3.2 | 25.5 ± 2.0 |
| **900 mg/kg** | 28.8 ± 2.4 | 28 ± 2.8 | 26 ± 1.7 | 25.7 ± 2.5 | 25.7 ± 3.3 | 25.4 ± 3.5 | 25 ± 2.1 |

**Table- S1** Body weights of vehicle fed (control) and MEF_M2_-treated breast cancer bearing mice

**Table-S2** Body weights of MEF_M2_ treated/control mice in Sub-acute toxicity study (28 days)

|  | **Day 0** | **Day 5** | **Day 10** | **Day 15** | **Day 20** | **Day 25** | **Day 28** |
| --- | --- | --- | --- | --- | --- | --- | --- |
| **Control** | 24 ± 0.2 | 24 ± 0.7 | 24.2 ± 0.35 | 24 ± 1.1 | 24 ± 0.9 | 24.2 ± 1.2 | 24.6 ± 0.6 |
| **Treated** | 24.2 ± 0.3 | 24.6 ± 0.5 | 25 ± 0.4 | 26 ± 0.8 | 26.5 ± 0.5 | 27.3 ± 0.3 | 27.5 ± 0.5 |

**Table-S3** Body weights of MEF_M2_ treated/control mice in Sub-Chronic toxicity study (90 days)

| **Female** | **Control** |  | **Day 0** | **Day 10** | **Day 20** | **Day 30** | **Day 50** | **Day 70** | **Day 90** |
| --- | --- | --- | --- | --- | --- | --- | --- | --- | --- |
|  |  | **Avg** | 24 | 24.25 | 24 | 24.4 | 25.4 | 24.8 | 25 |
|  |  | **St. dev** | 1.25 | 1.25 | 3.39 | 1.14 | 1.14 | 2.28 | 2.67 |
|  | **Treated** | **Avg** | 24.5 | 24.6 | 24.4 | 26.5 | 27 | 27.5 | 27.5 |
|  |  | **St. dev** | 2.3 | 2.4 | 2.7 | 2.3 | 2.7 | 3.5 | 2.9 |
| **Male** | **Control** | **Avg** | 22.7 | 22.5 | 21.5 | 22.5 | 22 | 23 | 24.6 |
|  |  | **St. dev** | 3.5 | 3.53 | 4.95 | 3.5 | 1 | 3 | 5.1 |
|  | **Treated** | **Avg** | 23.7 | 23.4 | 24 | 24.2 | 24.5 | 25 | 26.6 |
|  |  | **St. dev** | 2.73 | 3.61 | 2.91 | 3.61 | 4.69 | 3.2 | 3.98 |

**Table-S4** Body weights of MEF_M2_ treated/control mice in chronic toxicity study (180 days)

|  |  |  | **day 0** | **day-20** | **day-40** | **day-60** | **day-80** | **day-100** | **day-120** | **day-140** | **day-160** | **day-180** |
| --- | --- | --- | --- | --- | --- | --- | --- | --- | --- | --- | --- | --- |
| **MALE** | **Control** | **Average** | 21 | 21.7 | 22.2 | 22.36 | 22.6 | 23 | 23 | 23.36 | 23.5 | 23.5 |
|  |  | **SD** | 3 | 3.2 | 2.3 | 2.1 | 2.6 | 3.9 | 3.2 | 3.7 | 3.5 | 4.9 |
|  | **Treated** | **Average** | 21.5 | 22 | 24 | 25 | 26.3 | 26.5 | 27.5 | 27.6 | 27.1 | 27.75 |
|  |  | **SD** | 3.42 | 3.9 | 3.58 | 3.78 | 4 | 4.19 | 5.24 | 4.82 | 5.1 | 4.2 |
| **Female** | **Control** | **Average** | 17.8 | 18 | 18.7 | 19.4 | 19.4 | 20.4 | 20.5 | 20.6 | 20.7 | 20.8 |
|  |  | **SD** | 3 | 4.41 | 3.4 | 3.28 | 2.0 | 3.0 | 3 | 4.0 | 2.3 | 3.1 |
|  | **Treated** | **Average** | 17.5 | 18.5 | 20.7 | 20.8 | 21.8 | 22.4 | 22.6 | 22.75 | 23 | 23.5 |
|  |  | **SD** | 2.3 | 4 | 2.28 | 2.16 | 2.17 | 2.6 | 2.2 | 1.7 | 2.06 | 1.9 |

**Figure S1**

**Figure S1** *Graphical representation of body weights of control and three treated groups of breast cancer-bearing mice.*

*Error bars in all graphs representing the mean ± SD. There is a significant difference (p<0.05) between control and treated groups in their tumor sizes and body weights.*
